# Salutogenic health promotion program for migrant women at risk of social exclusion

**DOI:** 10.1186/s12939-019-1032-0

**Published:** 2019-09-03

**Authors:** A. Bonmatí-Tomas, M. C. Malagón-Aguilera, S. Gelabert-Vilella, C. Bosch-Farré, L. Vaandrager, M. M. García-Gil, D. Juvinyà-Canal

**Affiliations:** 1 Faculty of Nursing, University of Girona, Emili Grahit, 77, 17071 Girona, Spain; 2 Health and Health Care Research Group, University of Girona, Emili Grahit, 77, 17071 Girona, Spain; 3 European Training Consortium-Public Health and Health Promotion, Emili Grahit, 77, 17071 Girona, Spain; 4 Department of Social Sciences, Heath and Society, Wageningen University & Research, Hollandseweg 1, Wageningen, KN 6706 The Netherlands; 5 Vascular Health Research Group, Unitat de Suport a la Recerca Girona, Institut Universitari d’Investigació en Atenció Primària Jordi Gol (IDIAPJGol), Girona, Spain; 6 Information System for Research in Primary Care (SIDIAP), Institut Universitari d’Investigació en Atenció Primària Jordi Gol (IDIAPJGol), Barcelona, Spain; 7 Chair of Health Promotion, University of Girona, Emili Grahit, 77, 17071 Girona, Spain

**Keywords:** Salutogenesis, Migrant women, Health promotion, Self-esteem, Perceived stress, Quality of life

## Abstract

**Background:**

Migrant women at risk of social exclusion often experience health inequities based on gender, country of origin or socioeconomic status. Traditional health promotion programs designed for this population have focused on covering their basic needs or modifying lifestyle behaviors. The salutogenic model of health could offer a new perspective enabling health promotion programs to reduce the impact of health inequities. This study evaluated the effectiveness of a salutogenic health promotion program focused on the empowerment of migrant women at risk of social exclusion.

**Methods:**

A four-session salutogenic health promotion program was conducted over a period of 6 months. In a quasi-experimental pre-test post-test design, an ad hoc questionnaire was administered to 26 women to collect sociodemographic data, together with 5 validated instruments: Antonovsky’s Sense of Coherence (SOC-13), Duke-UNC-11 (perceived social support), Quality of Life Short Form-36 (SF-36), Rosenberg’s Self-Esteem Scale, and the Cohen et al. Perceived Stress Scale (PSS-10). Descriptive analysis and multiple linear regression models were performed. Statistical tests were considered significant with a two-tailed *p* value < 0.05.

**Results:**

Participants had a low initial SOC-13 score (60.36; SD 8.16), which did not show significant change after the health promotion program. Perceived social support (37.07; SD 6.28) and mental quality of life also remained unchanged, while physical quality of life increased from 50.84 (SD 4.60) to 53.08 (SD 5.31) (*p* = 0.049). Self-esteem showed an increasing trend from 30.14 (SD 4.21) to 31.92 (SD 4.38) (*p* = 0.120). Perceived stress decreased from 20.57 (SD 2.91) to 18.38 (SD 3.78) (*p* = 0.016). A greater effect was observed at the end of the program in women with lower initial scores for SOC-13 and quality of life and higher initial scores of perceived stress.

**Conclusions:**

The health promotion program reduced perceived stress, increased physical quality of life and showed a trend toward increased self-esteem, especially among migrant women with multiple vulnerability factors. The salutogenic model of health should be considered as a good practice to apply in health promotion programs and to be included in national policies to reduce health inequity in migrant populations.

**Electronic supplementary material:**

The online version of this article (10.1186/s12939-019-1032-0) contains supplementary material, which is available to authorized users.

## Background

Migration is a dynamic individual or group process that affects personal relationships at the point of origin and the destination. It is therefore not merely a spatial or temporary movement of people, but a process of necessary cultural readaptation in the new environment [1]. The migrant population can be described as a very heterogeneous group with a great diversity of life experiences closely related to the migration process [2].

In recent decades, global migration has increased considerably [3]. According to Eurostat data, 36.9 million of the migrants to Europe in 2017 were born outside of EU-28 countries. During that same year, Spain counted approximately 4.4 million migrants [4]. In Catalonia, foreign-born legal residents were less than 2.5% of the population in 2001; in 2018, they constituted 13.78% of the Catalan population (approximately 1 million people).

In the region of Girona, the number of migrants in 2017 was about 140,000, almost 18% of the population. Among these, 51.8% were between 24 and 50 years old. The migrant population consisted of 48.2% women. In three specific villages, the percentage of migrants is even higher, around 40% of the population. The majority of the migrant communities in the region are Moroccan (36,467 people, almost 25% of the total population), followed by Hondurans (9595 people, 6.8%) and Gambians (7155, 5%) [5].

### Stressors of migration

Migration can negatively affect physical, mental and emotional health [6, 7]. It has a stressful effect on people because it begins with the hope of a new and better life but experiences at the new destination often cause unexpected loss and culture shock [8, 9]. Migrants must cope with these stressors until they can regain a sense of balance or adapt to the new situation [9, 10]. For these reasons, migrants are considered a vulnerable population [11].

Although migrant populations are among those most affected by health inequities, few health programs and policies focus specifically on reducing inequities in these vulnerable populations [12–14]. Migrant women are especially at risk due to gender discrimination, stigmatization, and lack of resources [15]. Gender is a social determinant of health that triggers differences in the socialization of women and men. This implies distinctive values, attitudes, and behaviors that lead to gender-based differences in access to resources and jobs [16]. Moreover, migrant women usually work at home, so their work is unpaid, ignored, and not recognized by others [17].

In addition, they suffer from the stigmatization or discrimination [18, 19] to which the migrant population is especially vulnerable [20]. Migrant individuals are often targeted for their differentiating features, ethnic traits or cultural behaviors [21]. It is known that self-esteem helps to cope with stigmatization [22] and that a high level of self-esteem and self-confidence help to reduce perceived stress, allowing the individual to choose the most effective coping strategies [23]. A high level of self-esteem together with strong social support reduces vulnerability to stressors [24, 25], and social support is a protective determinant for adults to adapt to a new environment [26] and to cope with stressful situations [27].

Finally, lack of resources in migrant populations causes difficult life conditions and limits access to opportunities. In Spain, the unemployment rate is 19% in the native population, while it is 35% among migrants [28].

### Health promotion programs based on the salutogenic model of health

The aim of health promotion programs for migrant individuals are generally focused on covering their basic needs, such as food and housing [29], or on modifying lifestyle behaviors [30, 31]. Although necessary, these programs are not sufficient to overcome health inequities. New approaches and new strategies are needed. In Spain, the strategic goals of the national program in prevention and health promotion include a commitment to tackle health inequity and to facilitate the health empowerment of individuals [32]. Moreover, the Spanish Commission to Reduce Social Inequalities in Health was created in 2008 specifically to develop a nationwide proposal for interventions to reduce health inequities [33]. In accordance with national policy and strategies, the present study introduced the salutogenic model developed by Aaron Antonovsky into a health promotion program in an effort to explore new approaches that can be effective in reducing health inequities.

Antonovsky’s salutogenic model is based on a health-disease continuum between dis-ease and ease. Salutogenesis focuses on the origins of what creates health (strengths and resources) and focuses attention toward the development of health [34]. In contrast, the usual pathogenic approach considers health and disease as two mutually exclusive states and focuses on the risk factors associated with disease [35]. Salutogenesis suggests that stressors are common in daily life, generating tensions that can cause a landslide of health consequences if the individual succumbs to them, or a positive health effect if the tensions are overcome [34].

The key elements in Antonovsky’s theory of salutogenesis are generalized resistance resources (GRRs) and sense of coherence (SOC). GRRs are defined as “a property of a person, a collective or a situation which, as evidence or logic has indicated, facilitates successful coping with the inherent stressors of human existence”. SOC is defined as “the ability to understand the overall situation and the capacity to use available resources” [35]. Antonovsky described three SOC dimensions: comprehensibility, manageability and meaningfulness. Comprehensibility represents the person’s capacity to transform stimuli into information about what is being experienced or perceived as meaningful, orderly, consistent, structured, and clear. Manageability refers to the individual’s perception of the available resources and their adequacy to meet daily demands. Meaningfulness indicates the extent to which a person feels that life makes sense emotionally and is sufficiently motivated to put effort into confronting problems and difficulties [36].

The aim of health promotion programs with a salutogenic model of health is to increase the internal and external awareness of the GRRs and enhance the ability to use them in daily life [37] in order to improve health. These programs are focused not only on minimizing (disease) risks, but also on strengthening the existing GRRs of each participant, thus facilitating the participant’s progress towards health as described by Antonovsky. This study explored the effectiveness of a salutogenic health promotion program for migrant women at risk of social exclusion.

## Methods

### Study design

A quasi-experimental pre-test post-test study was designed. The intervention consisted of a salutogenic health promotion program, carried out at a job placement program of *Caritas*, the international Catholic Charities organization that works with people who are unemployed, homeless and/or who are migrants. The study took place in Girona (Spain), beginning in January 2015 with participant recruitment and ending in February 2016 with post-test data collection.

### Description of the salutogenic health promotion program

The main objective of this salutogenic health promotion program was to improve awareness of GRRs and increase self-esteem, physical and mental quality of life (QL), and SOC in the study population, and decrease their perception of stress. For this reason, program activities were planned with the aim of raising questions and encouraging the participants to help each other identify their own resources through the contributions of the group discussions. The role of the facilitator of the sessions was to enable the women to discover their GRRs.

**Fig. 1:**
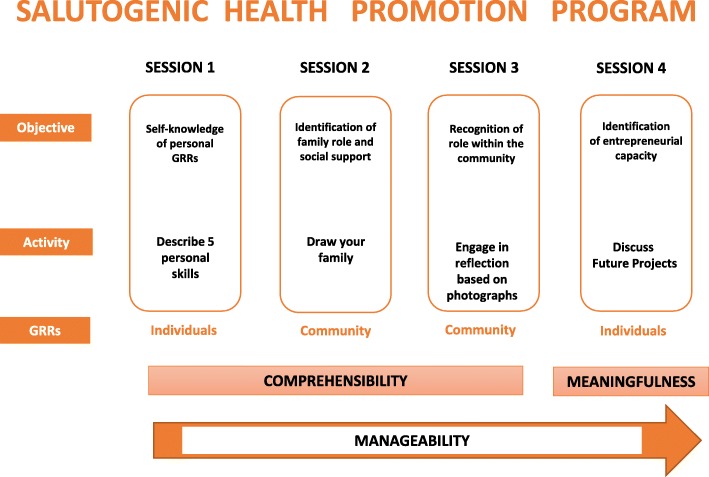
Description of the health promotion program and its relation with the salutogenic model of health

This program consisted of 4 group sessions, each lasting 2 h for a total of 8 h, a duration similar to analogous programs in other studies [38] (Fig. 1). The specific activities carried out in each session were as follows:

- **SESSION 1:** Improving self-knowledge of individual GRRs. As an individual activity, each participant was encouraged to describe 5 qualities about herself. Afterwards these qualities were shared with the rest of the group.
- **SESSION 2:** Identifying community GRRs, including self-recognition of one’s own role in the family and family social support. Two activities were developed. The first consisted of participants drawing a picture of their families. In the second activity, different situations were presented to enable participants to recognize their role and their available support in the family (e.g. who takes care of family members when they are ill? Who is in charge of the household shopping? Who attends the parent-teacher meetings? Who is in charge of the paperwork, of paying the household bills? In case you get ill, who would you ask for help? Who do you think would help you? In case of economic hardship, to whom would you explain it? Who do you think would help you?).
- **SESSION 3:** Exploring the role of each participant in her community; further identifying community GRRs. The participants were asked to take pictures that characterized what was important in their daily life as members of a community. These pictures were presented and explained to the rest of the group.
- **SESSION 4**: Building individual capacity to complete a personal project. A text about a woman entrepreneur was read and the meaning of entrepreneurship was discussed. In the end, each participant explained her own goals for the short, medium and long-term future.

Factors such as limited command of the language, low literacy level and the different cultural backgrounds of the participants created difficulties during the development of the study, both in the data collection phase and in designing the health promotion program. In order to reduce these difficulties, a facilitator with training in questionnaire administration was included on the research team. In addition, a good practice guide was created together with the technical team of *Caritas* to help both the facilitator of the questionnaire process and the session leader. As needed, participants were given more time to complete the questionnaire and the duration of each session was extended.

### Study population

Participants in the study were migrant women at risk of social exclusion who were participating in the *Ecosol* and *Avancem Santa Clara* programs of *Caritas Diocesana*. The 6-month programs focus on strengthening professional skills through a personalized itinerary, with the aim of helping these women join the job market. Participants were divided into 4 groups of 7 women each. The groups were intentionally heterogeneous, with participants of different ethnic origins, religious and cultural backgrounds, and languages.

All participants met the following inclusion criteria and consented to participate in the study: migrant women, older than 18 years, and at risk of social exclusion. Risk of social exclusion was assessed by the *Caritas* Institution according to the following criteria: communication difficulties, lack of work experience, lack of school education, single parenthood, family breakdown, subsistence or precarious employment, poverty, deprivation of freedom, lack of legal documents, isolation, cultural distance, illness, disability, lack of motivation, work habits not established, substandard housing, difficulties in having geographic and work-schedule flexibility, and unemployment. Participants had to meet at least 6 of these criteria to be considered at risk of social exclusion.

### Independent variables and study outcomes

The effectiveness of the intervention was measured by comparing the results of a questionnaire before and after the 6-month salutogenic health promotion program.

The questionnaire included the following information:

- Independent variables:
  - Sociodemographic data: age, country of birth, background, geographic area, years of education.
  - Migration history and current situation: number of years away from home country, number of years living in Spain, marital status, number of offspring, current cohabitation status, dependent family members, proportion of time dedicated to the family.
  - General data: current employment status, self-perception of the current socioeconomic situation, importance of spirituality in her life.
- Dependent variables:
  - Sense of coherence (SOC-13), using the Spanish version of a scale created by Aaron Antonovsky [39] that has been validated for use in the population of Spain [40, 41].
  - Duke-UNC-11, assessing perceived social support using the Spanish version of the instrument developed by Bellon Saameno et al. [42].
  - Quality of Life, Short Form-36 (SF-36 v.2), adapted and validated by Vilagut et al. [43].
  - Rosenberg’s Self-Esteem Scale, using the validated Spanish version of Martiín-Albo [44].
  - The validated Spanish version of the Perceived Stress Scale (PSS-10) developed by Cohen et al. [45].

### Data analysis

A descriptive analysis of the sociodemographic variables was carried out. The binary relationship between variables was calculated using Pearson’s chi-square test/Pearson’s correlation, Student t-test and one-way ANOVA test. The bivariate effectiveness of the program was evaluated by Student paired t-test or Wilcoxon test. Normal distribution was assumed with the Kolmogorov-Smirnov and Shapiro-Wilk tests.

A sequential multiple regression analysis was used to predict the factors associated with the effectiveness of the program. Initially, the model included as independent variables the factors with published evidence of statistically significant bivariate association with the selected program.

The *Statistical Package for the Social Sciences* (SPSS) v.20.0 for Windows was used for data processing and analysis. The level of significance (p) was established as *p* < 0.05.

## Results

From the 30 women enrolled in the programs *Ecosol* and *Avancem Santa Clara* offered by *Caritas Diocesana* in Girona, 28 started the health promotion program. One of them quit after the second session and another missed the final session. The sociodemographic data of the 26 participants who completed the study are summarized in Table 1. The majority of them migrated directly to Spain, are married, have children, and are living with family or relatives who are dependent in some way. They have a basic level of education, place high value on spirituality or religious faith in their daily life, and report a bad or very bad perception of their economic situation and a high level of spirituality.

**Table 1:** Socio-demographic profile of the participants

	TOTAL (*n*=26)	%
Age		
18-35 year old	11	42.3
> 35 year old	15	57.7
Country/Region of birth		
Morocco	10	38.5
Sub-Saharan Africa	10	38.5
Latin American Countries	6	23.0
Rural/urban origin		
Rural	4	15.4
Urban	22	84.6
Years outside the country of birth		
0-10 years	12	46.2
> 10 years	14	53.8
Residency Background		
Migrated directly to Spain	23	88.5
Migrated to other countries	3	11.5
Years of schooling		
< 7 years	6	23.1
7-11 years	13	50.0
> 11 years	7	26.9
Importance of spirituality		
Low (0-7)	4	15.4
High (>7)	22	84.6
Marital status		
Single/Separated/Divorced	8	30.8
Married	18	69.2
Number of children		
0	2	7.7
1 and 2 children	9	34.6
3 or more children	15	57.7
Current cohabitation status		
With family or relatives	22	84.6
Without family nor relatives	4	15.4
Dependant family members		
No	1	03.9
Yes	25	96.1
Daily proportion of time dedicated to family		
None to quite a lot (0-5)	13	50.0
Much time to all my time (5-10)	13	50.0
Current employment status		
Paid work outside the home ^a^	20	76.9
Unemployment	6	23.1
Perceived socioeconomic status		
Good	10	38.5
Bad or very bad	16	61.5

NOTE: The variables are expressed with absolute frequency and the percentage

^a^This remuneration was the monetary contribution they received monthly from Caritas Institution for the work done in the programs in which they participate, which did not exceed in any case the 300€

### Associations between dependent variables and sociodemographic data

The results of the pretest questionnaire according to the social demographic data are shown in Additional file 1. On one hand, women from Sub-Saharan African countries had higher self-esteem compared to women from Morocco and Latin-American countries. On the other hand, participants who reported their socioeconomic situation as “bad” or “very bad” had higher levels of perceived stress than participants who considered their socioeconomic status to be “good”. Furthermore, participants from rural environments had higher scores in the mental dimension of quality of life, compared to those from urban areas.

### Effectiveness of the salutogenic health promotion program

After their participation in the health promotion program, participants showed a remarkable decrease in the SOC dimension of comprehensibility and in their perceived stress [see Table 2]. They also had an increased physical QL and showed a trend toward increased self-esteem.

**Table 2:** Mean differences and distribution of the variables of interest before and after the health promotion program

	Mean PRE	SD	Mean POST	SD	∆ mean Post-Pre	p Kolmo-gorov-Smirnov	p Shapiro- Wilk	p Student T
SOC	60.36	8.16	59.81	8.16	-0.55	0.2	0.876	0.850
Comprehensibility	22.71	4.28	21.15	3.98	-1.56	0.000	0.000	0.018*
Manageability	18.18	3.76	18.85	3.08	0.67	0.200	0.209	0.498
Meaningfulness	19.46	4.09	19.81	3.73	0.35	0.023	0.147	0.569*
Self-esteem	30.14	4.21	31.92	4.38	1.78	0.200	0.543	0.120
Perceived Stress	20.57	2.91	18.38	3.78	-2.19	0.200	0.394	0.016
Social Support	37.07	6.28	37.08	5.56	0.01	0.200	0.375	0.782
Physical QL	50.84	4.60	53.08	5.31	2.24	0.200	0.216	0.049
Mental QL	46.00	5.90	45.62	7.13	-0.38	0.200	0.589	0.697

NOTE: Quantitative variables are expressed as mean and standard deviation (SD)

Changes in the dependent variables between the pretest and posttest in relationship with the sociodemographic data are shown in Additional file 2. A greater increase in SOC can be observed in the women from Latin-American countries who were not living within a family setting at the moment of the study. Mean values for self-esteem increased more among participants from Latin-American countries, compared to those from Sub-Saharan African countries. In contrast, a decrease in self-esteem was observed among the women from Morocco. Participants with responsibility for dependent family members experienced a decrease in the mean value of perceived stress. Women younger than 35 years increased their mean physical QL score, while the older participants showed a decrease. In contrast, the younger women reported a decrease in the mean quality of mental health, while participants older than 35 years reported an increase.

### Multiple correlations

Findings presented in Additional file 3 showed that all variations in mean values were inversely related to participants’ own initial value. In addition, an inverse relationship between the change in perceived stress and initial mental health QL was observed (− 0.426; *p* < 0.05). Thus, the decline in perceived stress was greater in women who initially reported a lower mental QL. On the other hand, the initial physical QL scores (0.466; *p* < 0.05) were positively correlated with changes in self-esteem.

### Multiple regression analysis

**Fig. 2:**
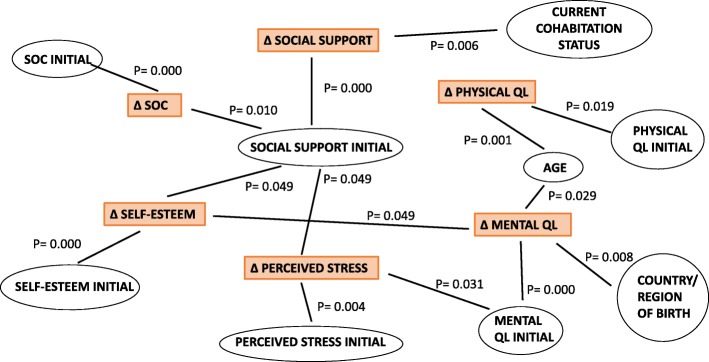
Variables showed relationship with the change of SOC, Self esteem, Perceived Stress, Social Support, Physical QL and mental QL

Multiple regression analysis was carried out to examine the relationship between the change in each construct and the various potential predictive factors. Additional file 4 summarizes the initial model (model 1), which compiled all independent variables, and the final model (model 2), which assembled the variables having a statistically significant correlation with the change in each construct after the health promotion program [see Fig. 2].

The health promotion program contributed to a greater increase in SOC in participants with a higher social support score and lower SOC at baseline, compared to the mean. The greatest change in perceived social support was observed in participants with a low initial score who were living within a family setting.

Higher perceived social support and mental QL at baseline were correlated with an increase in self-esteem at follow-up. In contrast, the higher the initial self-esteem values, the less change was observed. Eventually, a greater change in perceived stress was noted in participants with lower initial values for perceived stress, mental QL, and social support.

In reference to the quality of mental and physical health, greater change in physical QL was detected in younger women (< 35 years old) and in participants with lower initial physical QL scores. In contrast, greater change in mental QL was observed in older women and participants coming from Latin-American countries.

## Discussion

The salutogenic health promotion program was associated with a significant reduction in perceived stress, an increase in physical QL, and a tendency to increase self-esteem of the participants. Furthermore, this type of health promotion programs was more effective in women with lower initial scores in SOC, perceived stress, and mental and physical QL.

The lower scores in mental QL [46] and self-esteem detected in the study participants, compared with the mean for women born in Spain, are consistent with the results of other studies [47]. Some authors have connected these findings with the triple discrimination that migrant women at risk of social exclusion experience due to their gender, cultural, and social status [48].

The activities of the health promotion program enabled participants to self-recognize not only their internal GRRs, but also those in their community and broader environment (external GRRs). In this sense, awareness of the GRRs promotes increased self-esteem, as previously reported [35]. Our results are consistent with the findings of an Australian study using a health promotion program and objectives similar to the present study [49].

In contrast, self-awareness of GRRs did not support an increase in participants’ SOC, which might be attributed to two explanations. The first, as suggested by other authors, is that the SOC remains constant for people older than 30 years [36]. The second is that this specific health promotion program did not include activities that develop the ability to use the GRRs in daily life.

Apart from this, the lower the initial parameters of perceived stress, self-esteem, mental and physical QL, and social support, the higher the observed changes in these parameters. This suggests greater effectiveness of salutogenic health promotion programs among women living in situations of greater vulnerability. Consequently, such programs would be especially effective in reducing health inequities among the most vulnerable population groups. This is especially relevant because health interventions that are not specifically designed to benefit vulnerable populations may actually increase inequities [50]. In addition, this program was aligned with current national policy guidelines in Spain [33] that prioritize this strategic goal for health programs designed for the most vulnerable populations.

The significant decrease in perceived stress we observed is consistent with the findings in other studies [51]. The program activities were focused on awareness of stress protectors such as social support and knowing one’s role in the family [52], which could explain these results.

Another aspect to take into account, given our study results, is the paradoxical increase in the physical but not in the mental QL of the participants after the program. Two factors might explain this observation: differing cultural concepts of “health” and the complexity and multidimensional definition of QL. In some cultures, mental health problems stigmatize the affected person [53]. As a result, people tend to “unconsciously hide” their mental discomfort, with a somatization into physical discomfort [54]. In this sense, the increased physical QL perceived by young adults such as our participants could be attributed to their improvement in self-esteem and reduction of stress, which they perceived as an improvement in their physical QL. Second, the inclusion of physical, psychic and social aspects, and also subjective and objective facets of each participant, in defining QL [55] makes it particularly difficult to assess the changes in each dimension separately.

This study had several limitations. The small sample size affected the statistical power of the analysis. Furthermore, the use of a convenience sample limited the external validity of the results; this bias was diminished with the inclusion of participants of different ages, cultures, and backgrounds. Another limitation was the quasi-experimental design without a control group, which made it impossible to compare the results achieved by this intervention with other types of interventions.

Despite these limitations, the present study has some strengths. One of them is the successful administration of a quantitative longitudinal questionnaire in a population of migrant women with high rates of illiteracy and general difficulties in communicating. These characteristics contribute to their exclusion from most quantitative studies. In turn, this exclusion reinforces the lack of effective health promotion programs for this population, thus perpetuating health inequities.

Another strength of this study is the introduction of the salutogenic model into the program design. This new model of health takes into consideration the capacity of people to empower themselves using their own GRRs, which often go unrecognized by themselves and by their environment. This new approach may enable a new societal perspective on vulnerable populations, a vision that treats the collective of vulnerable people as individuals with abilities and resources even though they temporarily have some needs to be met.

The present study results have important implications for policymakers. Based on previous evidence [56] that new models of health empowerment for migrants are needed to improve their long-term health and well-being, applying this new approach that encourages the abilities of disadvantaged individuals would go far beyond covering their basic needs. This would certainly reduce the stigmatization that vulnerable people experience today. In Spain, although health policy considers the reduction of inequities a priority in the political agenda, few health promotion initiatives have successfully integrated this new emphasis [33].

Further work is needed on long-term studies of vulnerable populations such as migrants. To study the effectiveness of the salutogenic health promotion programs, more longitudinal studies and interventional studies with representative samples are needed to support the adoption of policies to reduce health inequities. Additionally, new health promotion programs with the salutogenic model of health should be developed to enable participants not only to become aware of their own GRRs but also to use them in their daily life.

## Conclusions

The salutogenic health promotion program for migrant women at risk of social exclusion significantly reduced perceived stress, increased physical QL, and tended to increase self-esteem. The program was more effective in women with lower initial scores for SOC and QL and higher scores for perceived stress. Perceived social support is also a key factor in the empowerment of these migrant women.

The salutogenic model of health should be considered as a good practice to apply in health promotion programs and to be included in national policies to reduce health inequity in migrant populations.

## Additional files

Additional file 1: Initials SOC, social support, self-esteem, perceived stress and quality of life by socio-demographic data. (XLSX 16 kb)

Additional file 2: Variations of SOC, social support, self-esteem, perceived stress and quality of life by socio-demographic data. (XLSX 15 kb)

Additional file 3: Correlations between the variations of the variables before and after the intervention. (XLSX 10 kb)

Additional file 4: Models of the variation of SOC, social support, self-esteem, stress, physical quality of life and mental quality of life according to criteria of variables selection (*n* = 26). (XLSX 15 kb)

## Data Availability

The datasets used and/or analyzed during the current study are available from the corresponding author on reasonable request.

## Abbreviations

GRRs: Generalized Resistance Resources
QL: Quality of Life
SOC: Sense of Coherence
SPSS: Statistical Package for the Social Science
